# Necropsy findings, meat control pathology and causes of loss in semi-domesticated reindeer (*Rangifer tarandus tarandus*) in northern Norway

**DOI:** 10.1186/s13028-023-00723-9

**Published:** 2024-01-04

**Authors:** Torill Mørk, Henrik Isaksen Eira, Rolf Rødven, Ingebjørg Helena Nymo, Berit Marie Blomstrand, Sandra Guttormsen, Line Olsen, Rebecca Katherine Davidson

**Affiliations:** 1 Section of Food Safety and Animal Health Research, Norwegian Veterinary Institute, 9016 Tromsø, Norway; 2 Norwegian Nature Surveillance, Local Office, Finnmark Estate, 9521 Kautokeino, Norway; 3 Arctic Monitoring and Assessment Programme, AMAP, FRAM Centre, Hjalmar Johansens Gate 14, 9007 Tromsø, Norway; 4 Norwegian Centre for Organic Agriculture, 6630 Tingvoll, Norway; 5 Department of Arctic and Marine Biology, UiT, The Arctic University of Norway, Hansine Hansens Veg 18, 9019 Tromsø, Norway

**Keywords:** Arctic, Body condition, Disease, Infection, Parasites, Predator killed, Reindeer

## Abstract

**Background:**

Reindeer herding in Norway is based on traditional Sámi pastoralism with the animals free ranging throughout the year. The animals move over large areas in varying terrain and often in challenging weather conditions. Winter crises, such as difficult grazing conditions caused by icing or large amounts of snow, are survival bottlenecks for reindeer. Calves are especially vulnerable, and many may die from starvation during winter crises. Predation and starvation are the predominant narratives to explain losses, however, carcasses are difficult to find and often little remains after scavenging and decay. Documentation of the causes of death is therefore scarce.

**Results:**

In this study, we investigated the cause of reindeer mortality in Troms and Finnmark, Nordland and Trøndelag during 2017–2019. Necropsies (n = 125) and organ investigation (n = 13) were performed to document cause of death. Body condition was evaluated using visual fat score and bone marrow fat index. A wide range of causes of death was detected. The diagnoses were categorized into the following main categories: predation (n = 40), emaciation (n = 35), infectious disease (n = 20), trauma (n = 11), feeding related disease (n = 5), neoplasia (n = 4), others (n = 6) and unknown (n = 17). Co-morbidities were seen in a number of diagnoses (n = 16). Reindeer herders are entitled to economic compensation for reindeer killed by endangered predators, but a lack of documentation leads to a gap between the amount of compensation requested and what is awarded. An important finding of our study was that predators, during winter, killed animals in good as well as poor body condition. Emaciation was also shown to be associated with infectious diseases, and not only attributable to winter grazing conditions.

**Conclusions:**

This study highlights the importance of examining dead reindeer to gain knowledge about why they die on winter pasture. The work presented herein also shows the feasibility and value of increased documentation of reindeer losses during winter.

## Background

Reindeer herding in Norway is based on traditional nomadic pastoralism of the Sámi people whereby semi-domesticated reindeer (*Rangifer tarandus tarandus*) range freely throughout most of the year and feed on natural pastures [1]. In some areas, the percentage of animals lost during a herding year are high, however, losses and causes of death are often difficult to document given the free-ranging behaviour. According to the yearly reports from herders, predation is a major cause of losses in Norway [2–4].

Reindeer killed by the protected predators lynx (*Lynx lynx*), wolverine (*Gulo gulo*), brown bear (*Ursus arctos*), wolf (*Canis lupus*) and golden eagle (*Aquila chrysaetos*), as well as animals lost in serious accidents, are economically compensated by the Norwegian government [5, 6]. The compensation for predation loss is based on documentation of predator wounds on the carcasses, by specialists from Norwegian Nature Surveillance (SNO), combined with registration of predator presence in that area. Other relevant information, like tracks and traces from a battle between predator and prey in the area surrounding the carcass, is also evaluated [5].

Predators and scavengers will however, often have eaten most of the animal remains, and little will be available for investigation. Small and newborn calves are especially hard to recover and often completely devoured [7, 8]. During summer and early autumn, the degradation of carcasses is especially rapid due to higher environmental temperatures. This is also the time of year when animals are left the most unattended, except for the gathering and marking of calves. Winter is the season when carcasses are most likely to be found [8]. However, herders are dependent on good weather conditions, accessible terrain, as well as the human resources to search for lost animals.

Only about 5% of the carcasses inspected by SNO are documented as killed by predators [9] and according to official statistics for 2016–19, roughly 30% of the animals applied for as predator losses, were awarded compensation [10]. Non-predator causes of death are rarely registered. This lack of documentation of the cause of death, yields disagreement between reindeer herders and the public administration/management concerning the share of loss caused by predation and thus the rightful levels of compensation [7, 11].

Predators kill reindeer throughout the reindeer pasture regions [12–14]. Several studies have documented predators as an important cause of death, and certain areas have particularly high predation pressure [9, 14–16]. Previous studies have explored the potential connection between predation, population demographics and population dynamics of semi-domesticated reindeer in Norway [8, 17, 18]. The focus has been on animal weight, animal density, and climatic conditions compared to the number of animals lost to predation. These studies have concluded that high animal density, poor winter pastures, and poor body condition based on live weight or slaughter weight, to a large degree can explain the predation losses. These losses have therefore been regarded as compensatory, meaning that the animals killed by predators were the weakest in the herd, which would probably have died from starvation during winter had they not been killed by predators [8, 17, 18]. In Sweden and Finland, predatory losses are generally considered additive, meaning that they come in addition to other losses [19, 20]. However, this difference may possibly be in part due to differences in predator abundance, different reindeer population, and different herding systems [7, 21].

Winter is a bottleneck for survival, especially for calves [22]. During winters with large amounts of snow, with or without icecaps and icing of the pastures, large numbers of animals may die from starvation and the calving percentage may be lower than normal [23]. The Sámi reindeer herders use their traditional knowledge of snow cover, pasture ecology, as well as the natural behaviour and needs of the animals to minimise winter losses [24]. The opportunity to be flexible and move between pasture areas in order to be in the best place at the right time is essential and at the core of pastoralism [25]. Today there is, however, less flexibility due to management regulations and the loss of areas to anthropogenic structures and activities. In addition, climate change often leads to unfavourable winter conditions [1].

Due to the loss of pasture areas and more unstable winter conditions, the Norwegian reindeer herding system has been forced to adapt over the last few decades, with increasing use of supplementary feeding during winter, either with animals still free roaming or in enclosures [1]. Feeding is one way to cope with winter crises, to compensate for pastures with low nutritional value, or for the permanent loss of pastureland. The increased risk of infectious diseases when animals are kept in high density is described in older publications when the herds were kept closer together for larger parts of the year [1, 26–28]. Today, infectious diseases are becoming more prevalent in Finland and Sweden where feeding in enclosures is more common than in Norway [29, 30]. In Norway, there are still only a few reports of disease outbreaks directly connected to supplemental feeding [31, 32].

Climate change, with increasing summer temperatures, is also predicted to potentially increase diseases caused by temperature dependent parasites such as *Elaphostrongylus rangiferi* [33] and *Setaria tundra* [34], as well as the spread of vector borne diseases [27]. Trauma caused by animals falling off cliffs, avalanches, traffic accidents, etc. are likely to be underreported, and are known to be quite comprehensive in some regions [35, 36].

Reports of diseased animals or studies including necropsies, apart from the investigations for predator kills, are few [35, 37, 38]. Some studies have reported necropsy results [14, 15], however, it is unclear if these are full necropsies or mainly investigation of predation wounds. Therefore, the majority of disease cases are likely both underdiagnosed and underreported. This lack of systematic documentation of the cause of death in reindeer has led to disagreements between reindeer herders, public environmental managers, and researchers as to whether food limitation is the main indirect explanation for predator loss or if high predator pressure to a larger degree explains the losses [11, 39].

Studies increasing the understanding of other causes of loss, in addition to predation, are vital for improving the management of both predators and reindeer [40]. High losses are economically and psychologically burdensome for Sámi families as well as an important animal welfare issue requiring investigation and mitigation. In this study, we investigated causes of death by performing necropsies of semi-domesticated reindeer in northern Norway found dead or euthanized due to animal welfare considerations in the period 2016–2019. We also investigated animals found dead in slaughterhouse corals and organ samples with signs of disease lesions found during meat inspection. The aim of this work was to document and increase the knowledge of causes of loss in semi-domesticated reindeer.

## Methods

### Study area and samples

**Fig. 1:**
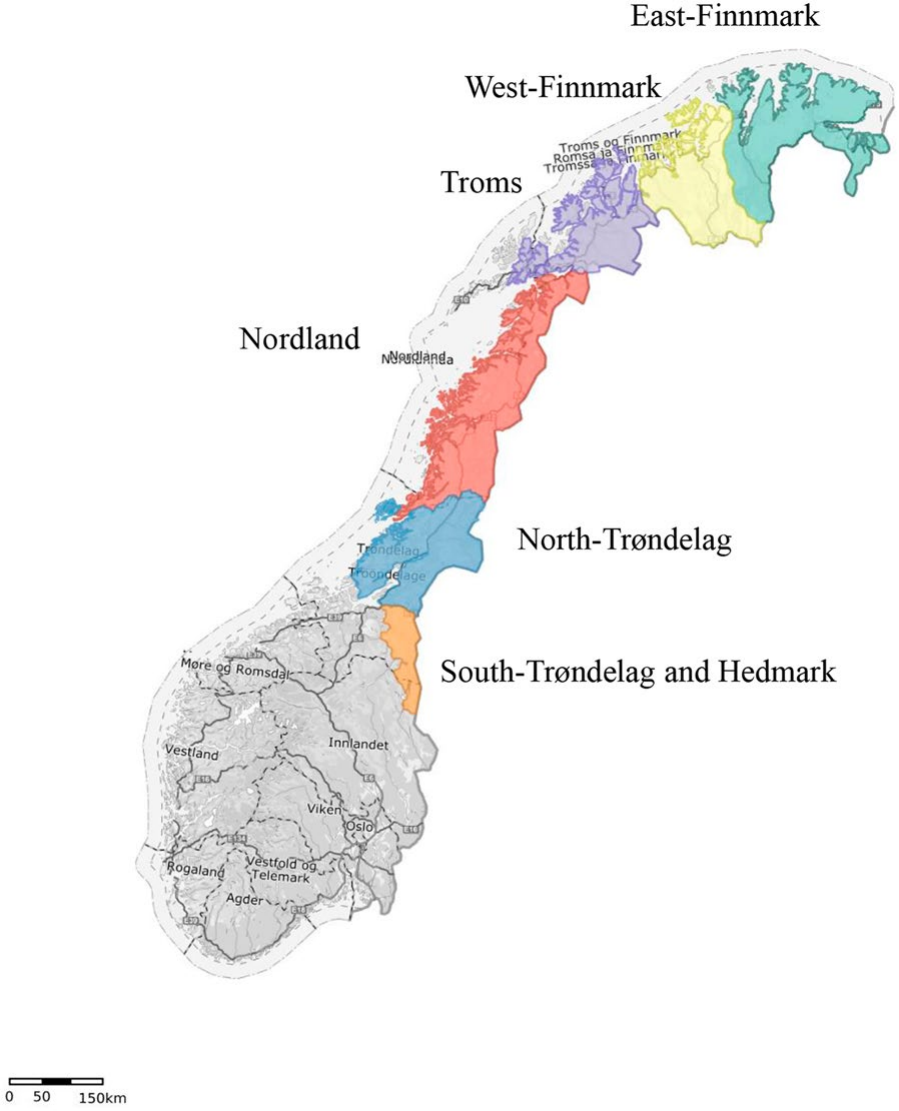
Map of Norway showing the six reindeer herding regions. The regions are divided into herding districts (administrative unit) and siidaes (families/working unit) (not shown). The study included material from all regions except South-Trøndelag and Hedmark but mainly from the three northernmost regions

All samples were from the three northernmost Norwegian counties; Troms and Finnmark, Nordland and Trøndelag (Fig. 1, Table 1) in the period December 2016–July 2019. In total, 125 carcasses were submitted for necropsy as either fresh (n = 57) or frozen (n = 68). Carcasses were either found dead (n = 117) or euthanised (n = 8).

**Table 1:** Total number of carcasses necropsied by region of origin and age group

Region	Newborn calves 0–3 months	Calves 4–11 months	Yearlings 12–24 months	Adults > 24 months	Total
Finnmark (19 districts)	8	25	3	28	64
Troms (6 districts)	13	16	6	23	58
Trøndelag (1 district)	0	3	0	0	3
Total	21	44	9	51	125

Carcasses were delivered by reindeer owners to the Norwegian Veterinary Institute in Tromsø (Troms, n = 52) and if suspected killed by predators, delivered to SNO in Tromsø (Troms, n = 6), Karasjok (East-Finnmark, n = 13), and Kautokeino (West-Finnmark, n = 46). Three carcasses from North-Trøndelag were animals that died showing clinical signs of brainworm infection (elaphostrongylosis) and were delivered to the Norwegian Veterinary Institute in Trondheim (n = 3). The Norwegian Food Safety Authority in Karasjok (East-Finnmark, n = 5), delivered carcasses of reindeer found dead during *ante mortem* inspection. Information regarding feeding history was available for 49 of the 125 carcasses.

All carcasses were categorized as newborn (0–3 months), calves (4–11 months), yearlings (12–24 months), and adults (> 24 months) (Table 1) according to tooth eruption pattern [41], time of year and body size. Age determination was carried out for 19 of the adults by counting the annuli in the cementum of a sectioned incisor tooth [42]. The age range in these individuals was 3.5–13.5 years.

The majority of animals died on winter pasture (n = 102). The newborn calves (n = 21) and one yearling died in spring/early summer, and one adult died in autumn.

The femur (*os femoris*) (n = 67, from 67 individuals) were also included in the study. These were collected from carcasses that died during the winter, but were not suitable for a full necropsy. The carcasses had, however, been previously investigated for predator wounds by SNO. The organ samples (n = 13, from 13 individuals) were from organs with disease lesions found during meat inspection or sampled during field necropsy from animals found dead in slaughter house corals during *ante mortem* control in autumn and winter (Table 2). These were examined for pathology (Table 2).

**Table 2:** Overview of additional reindeer organ samples examined by region of origin and age group

Region	Calves 4–11 months	Yearlings 12–24 months	Adults > 24 months	Unknown age	Total
Finnmark: Organ samples from *post mortem* control at slaughter	8	0	4	0	12
Finnmark: Bone samples after SNO evaluation	38	2	4	23	67
Nordland: Organ samples from *post mortem* control at slaughter	0	0	1	0	1
Total	46	2	9	23	80

### Necropsy

Carcasses were necropsied according to standard guidelines for *post mortem* examination at the Norwegian Veterinary Institute. Investigations for predator injuries were performed in cooperation with wardens from SNO in accordance with published national standards [43]. Samples for histology were fixed in 10% buffered formalin and embedded in paraffin. All tissues were routinely stained with haematoxylin and eosin (HE) and examined by light microscopy.

### Body condition

All carcasses were given a visual fat score based on the criteria given in Table 3. It was not always possible to assess every criterion due to loss of tissue. Femoral bone marrow samples were oven-dried to measure the fat percentage of bone marrow and provide a bone marrow fat index [44, 45]. Newborn calves (0–3 months) were not included in the bone marrow evaluation due to age-related ongoing erythropoiesis. The bone marrow fat index alone was used to evaluate the body condition from carcasses unfit for full necropsy (n = 67).

**Table 3:** Criteria for the visual fat score assessment of the carcasses of semi-domesticated reindeer

Visual fat score	0 Emaciated	1 Skinny/very poor)	2 Lean/poor	3 Average	4 Good/above average	5 Very good
Subcutaneous fat	None	None	Sparse to none	Sparse	Medium	Abundant
Fat depth (mm) at last lumbar vertebra	0	0	0	0	< 1 mm	> 1 mm
Omental fat	None	None	Sparse to none	Sparse	Medium	Abundant
Mesenterial fat	None	None	Sparse to none	Sparse	Medium	Abundant
Perirenal fat	None	Sparse	Medium	Medium	Abundant	Abundant
Fat “around” heart (epicardial fat)	None	Sparse	Medium	Medium	Abundant	Abundant
Bone marrow appearance	Gelatinous clear, red/orange	Gelatinous pink	Pink, incipient gelatinous	Pale pink, soft consistency	White, firm and fatty consistency	White, firm and fatty consistency

### Bacteriology

A standardised set of samples from the carcasses (liver, kidney, lung, and tonsil) were investigated with routine bacteriology. Other tissues were investigated if indicated by macroscopic pathological findings. Some carcasses and organs were not fit for bacteriology due to decay or absence of organs due to scavenging. Bacteriological cultivation was done according to standard operational procedures for detection of pathogenic bacteria at the Norwegian Veterinary Institute. Organ samples were sterilised with a gas flame. A small cut was made with sterile scalpel blades and agar plates were inoculated using sterile inoculation loops. All samples were incubated on blood agar at 5% CO_2_, on bromothymol blue lactose agar under aerobic conditions and on blood agar under anaerobic conditions at 37 °C. Plates were inspected after 18–24 and 48 h. Bacterial growth was examined and relevant/dominating colonies isolated and identified to genus or species level by Gram stain, biochemical tests (catalase, oxidase, indole) and API 20 test kits (BioMérieux, France).

### Parasitology

The presence and number of *Hypoderma tarandi* and *Cephenemyia trompe* larvae were registered and counted during necropsy. Modified McMaster and Baermann techniques [46] were used to detect and quantify nematode eggs, coccidian oocysts, and nematode larvae in the faeces. A subsample of the protostrongylidae larvae was examined for species identification.

### Diagnostic categories

**Fig. 2:**
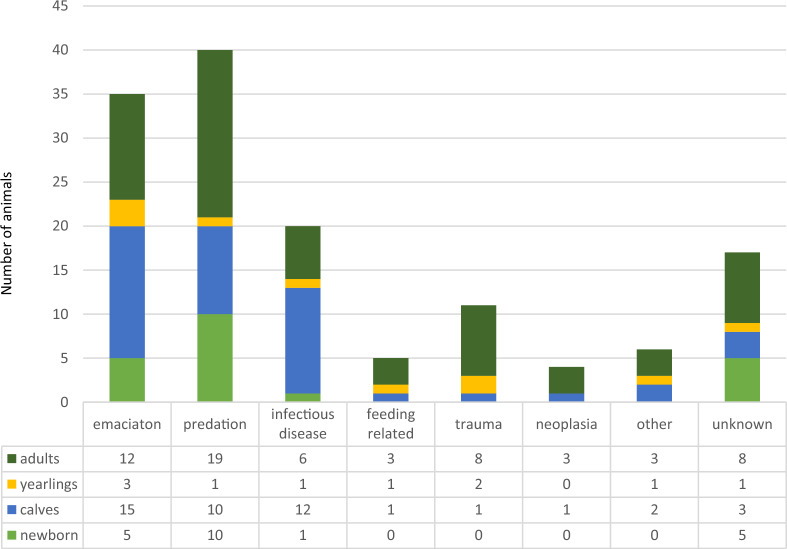
Distribution of age groups and main diagnostic categories. A histogram showing the diagnostic categories by animal age group for both necropsied carcasses and organ samples

The diagnoses were divided into eight categories based on the main findings; namely predation, emaciation, infection, feeding-related, trauma, neoplasia, other and unknown (Fig. 2).

### Statistics

This study was based on opportunistic sampling of carcasses, and it is therefore lacking an explicit pre-designed control group for comparisons. Hence, inferences are based upon differences among various causes of death and comparisons with referenced studies. Initially, the visual body fat score versus bone marrow fat index were compared using a one-way analysis with the whole data set, by age group and by sex, using JMP Pro 14.0.0 (SAS Institute Inc.). Pairwise student t-test was used to compare the means. Further analysis of the bone marrow fat index and visual fat score was carried out using R version 4.1.1 (R Core Team 2022).

For bone marrow data, absence of control groups made a standard logistic regression approach not feasible. Thus, descriptive histograms were used to evaluate the skewness of bone marrow fat index for reindeer killed by different predators.

Parasite burdens (counts of the number of eggs or oocysts per gram faeces) and bone marrow fat index showed proportions close to 0 or 100% with the confidence intervals being more asymmetric than transformation, which make linear models more prone to type I errors. To mitigate this a beta regression (R library betareg) [47] was used to compare these parameters with the different diagnostic categories. The parasite burdens were log-transformed (ln + 1) given their zero-inflated skewed distribution. The 95% confidence interval (CI) was calculated for disease prevalence in JMP Pro 14.0.0. A significance level of p = 0.05 was selected for all statistical methods.

## Results

### Body condition

**Fig. 3:**
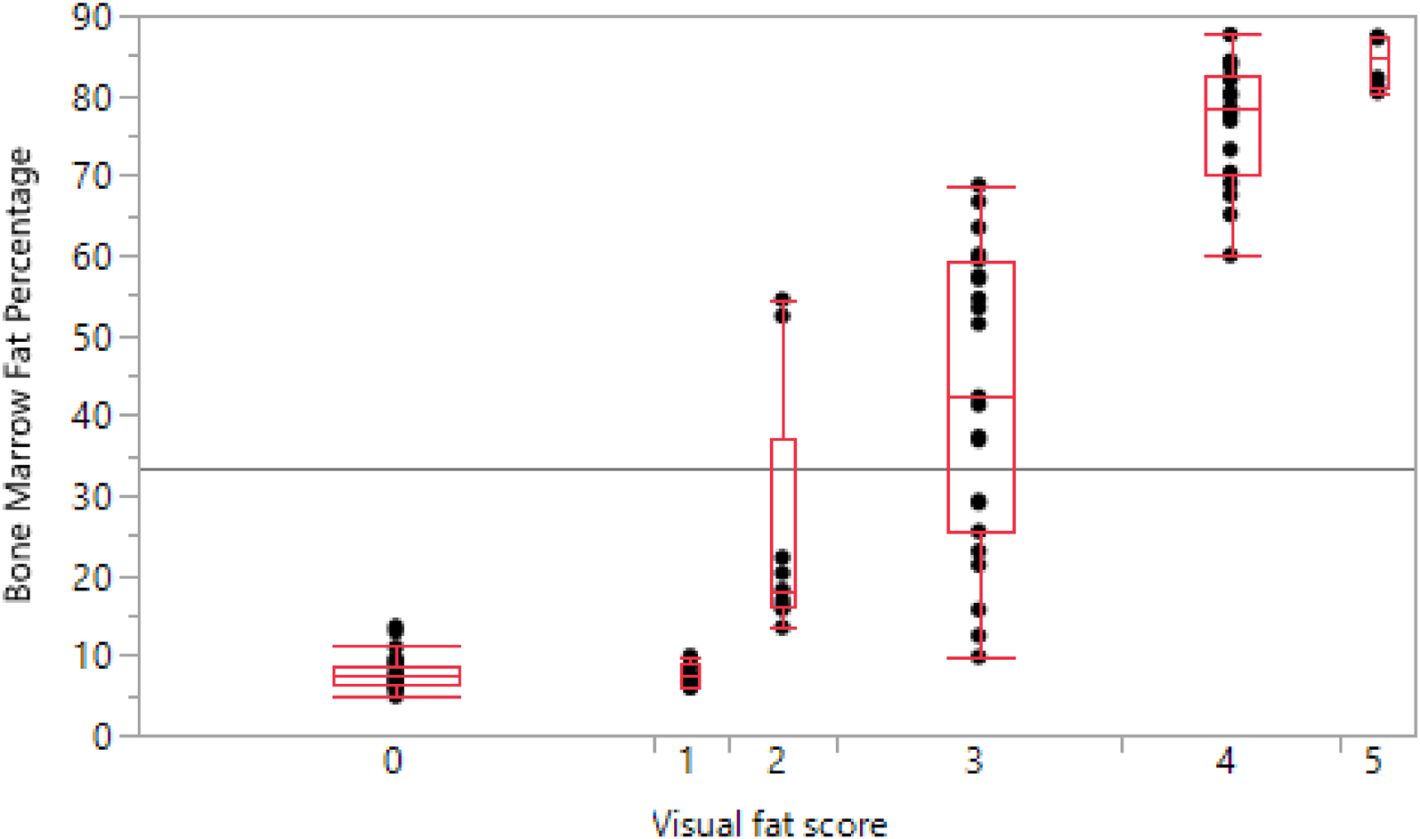
Bone marrow fat index and visual fat score. Quantile box plots of the bone marrow fat index by visual fat score (one-way analysis) for the reindeer submitted for necropsy, in 2016–2019, that had both body condition evaluations carried out (N = 104). Levels 0 and 1 do not have significantly different mean bone marrow fat indexes (%) but are significantly different to the other levels. Levels 2 and 3 are significantly different to all levels, and levels 4 and 5 are significantly different to all other levels but not to each other

Visual fat score revealed a broad range of body conditions (Fig. 3). There were no significant differences in the distribution of the visual fat scores or bone marrow fat index by the age (p = 0.22) or the sex of the animals (p = 0.33). Comparison of the two methods to estimate body condition showed significantly high levels of correlation (p < 0.001) between the bone marrow fat index and the visual fat score.

The bone marrow fat index explained 88% (Adjusted R squared value (AdjRSq) 0.879) of the variation in visual fat score in the whole data set. This held true for the bone marrow fat index in the three relevant age groups (excluding newborn calves); with bone marrow fat index in adults, yearlings and calves explaining 89% (AdjRsq 0.892); 95% (AdjRsq 0.946) and 95% (AdjRsq 0.945), respectively, of the visual fat score. The mean bone marrow fat index was not significantly different for animals classefied with visual fat score levels of 0 and 1 or between levels 4 and 5, however, they were significantly different to each other and to levels 2 and 3 (Fig. 3).

Despite this, there was considerable overlap in the bone marrow fat index range between animals in these two categories (Fig. 3).

**Fig. 4:**
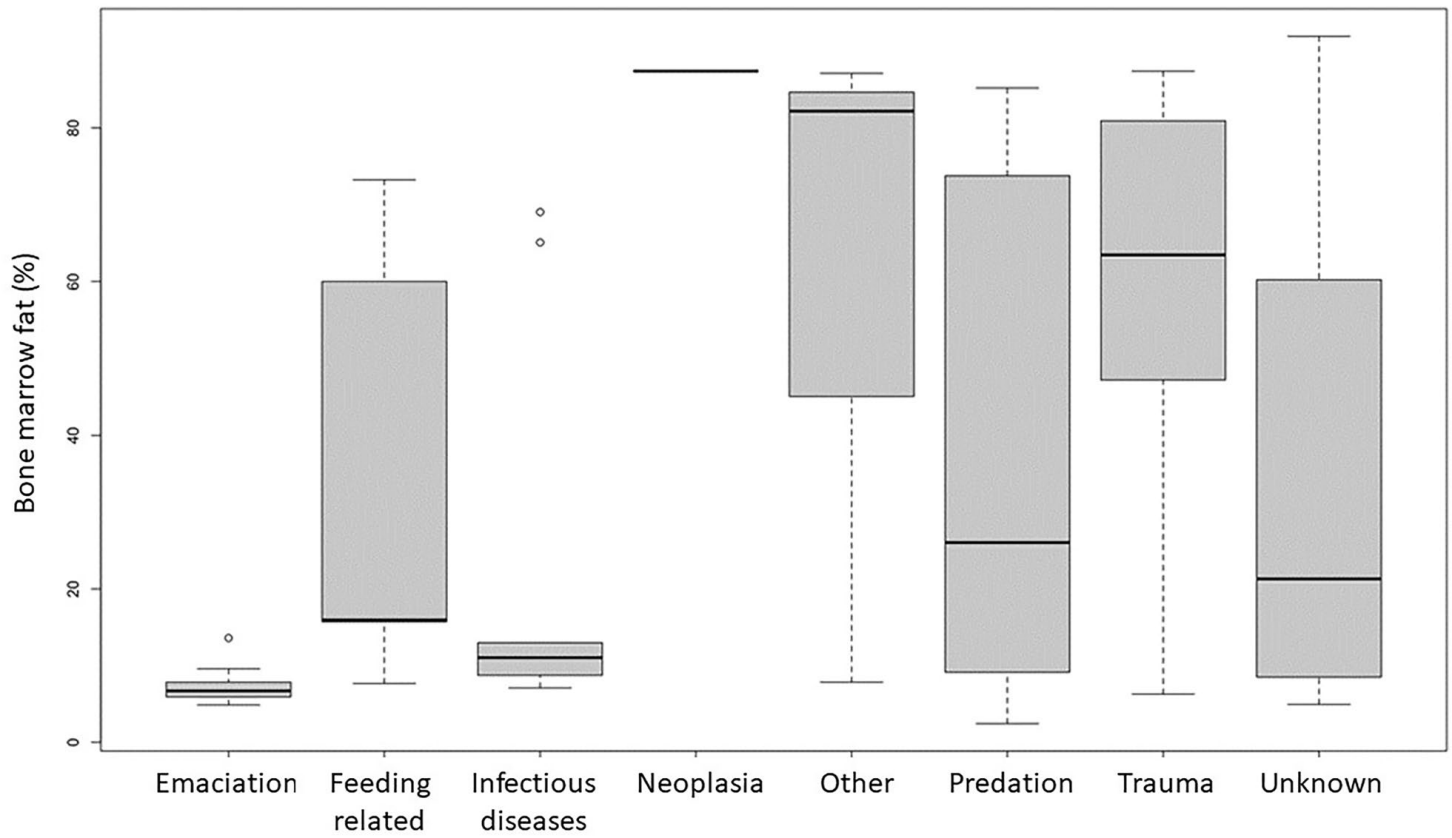
Quantile box plots of bone marrow fat index and main diagnostic category. Linear models using infections as reference level showed bone marrow index to be significantly lower in reindeer dying of infections compared to trauma (p = 0.002) or other causes (0.045). There was a tendency of a higher mean bone marrow percentage for animals killed by predators compared to those with infections (p = 0.072)

Linear models using infections as reference level showed bone marrow index to be significantly lower in animals dying of infections compared to trauma (p = 0.002) or other causes (p = 0.045). There was a tendency of a higher mean bone marrow percentage for animals killed by predators compared to those with infections (p = 0.072) (Fig. 4).

### Predation

The protected predator species found as cause of death in our study were golden eagle, wolverine, and lynx. We also registered predator kills from dogs, foxes and ravens.

**Fig. 5:**
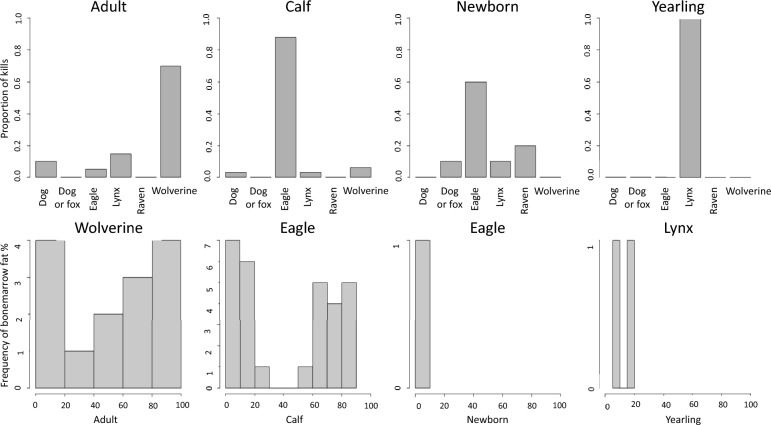
**a** (top row). Histograms showing the proportion of predator kills by the different predators in the different age groups.** b** Histograms showing the bone marrow fat index (BMFI) distribution of the animals killed by the dominant predator species for each age class

Predation was the most prevalent cause of death and was diagnosed in 32% of the carcasses submitted for full necropsy (40/125, 95% CI, 20.8–36.7) and in 40% of the carcasses not suitable for necropsy but examined for predator wounds by SNO and investigated for bone marrow fat index (27/67, 95% CI, 48.0–71.5). This gives a total number of 67/192 animals killed by predators (34.9% [95% CI 32.7–46.5]). There were significant differences in age class of animals killed by the different predator species (Fig. 5, Table 4).

**Table 4:** Overview of reindeer predated by predator and age group

Predator	Newborn	Calves	Yearlings	Adults	Unknown age	Total
Golden eagle	6	29	0	1	4	40
Wolverine	0	2	0	14		16
Lynx	1	1	2	3		7
Dog/fox	1			1		2
Raven	2					2
Total	10	32	2	19	4	67

The majority of animals in the newborn and calf age classes were killed by eagles (6/10 newborn and 29/32 calves killed by eagle), whilst lynx and wolverine more frequently predated on the older age classes (2/2 yearlings killed by lynx and 14/19 adults killed by wolverine). The adults and yearling results were combined for this analysis given that only two yearlings were diagnosed as killed by lynx. Four animals killed by eagle had no registration of age. The carcasses that had been predator killed were collected throughout the months October-July, but most of the carcasses (51/67) were received during winter, January-April, except for the newborn calves (9/67), which were killed in May, June and July.

Among carcasses predated, there were no significant trends relating to bone marrow fat index or visual fat index score. Newborn calves (due to the bone marrow fat variable not being suitable in this age class) and the yearling were excluded from this analysis. Calves and adults had individuals with either low or high percentage of bone marrow fat present in the predated group. Interestingly, in our study, animals with medium bone marrow fat index were under-represented in the predated group (Fig. 5, lower row).

### Emaciation

Emaciation was the second most prevalent diagnosis and was considered as primary cause of death in 28% [20.1–35.9] of the carcasses necropsied (35/125). The diagnostic criteria were loss of all body fat (fat index 0 and bone marrow fat index < 12) in addition to no findings of underlying causes of diseases or non-deadly traumas. Ten carcasses were identified as emaciated secondary to another primary diagnosis and were therefore not included here. Emaciation was diagnosed in 24% [5.6–42.0] of newborn calves (5/21), 39% [23.9–55.0] of calves (15/38), 39% [1.6–58.4] of yearlings (3/10), and 21% [10.7–32.2] of adults (12/56) (Fig. 2). These age group differences were not significant. The reindeer herders reported feeding 17 of the emaciated animals, from a few days to several months prior to death, whilst seven animals were not fed. Information regarding additional feeding was missing for the remaining animals.

### Infectious diseases

Infections were determined as the cause of disease or death in 14.2% [9.7–22.3 95% CI] (20/138) including both carcasses (n = 125) and organ samples (n = 13) from animals either found dead, euthanised because of disease or as a random finding in slaughtered animals. Infectious diseases were found mainly in calves (1 newborn calf, 12 calves, 6 adults, 1 yearling) in poor body condition (Figs. 2, 4).

Bacteriological investigation was carried out on samples from 106 individuals. *Pasteurella multocida* was the most prevalent bacterial species and isolated from 13 animals. Systemic pasteurellosis was diagnosed in four animals based on plentiful, pure or dominating growth of *P*. *multocida* from three or more organ samples. Systemic pasteurellosis was also diagnosed in combination with predator trauma in four additional cases. Bronchopneumonia related to *Pasteurella* spp. was diagnosed in three animals. In two cases the bacteria were identified with poor growth in a mixed culture. Other bacteria found as cause of infections were *Trueperella pyogenes*, *Streptococcus* sp., *Moraxella* sp., beta toxic *Staphylococcus* sp. and *Escherichia coli*.

Parasitic infections were registered from almost every carcass as secondary finding with the exception of three animals with elaphostrongylosis. However, the distribution varied with a strongly skewed zero-inflated distribution for faecal parasite analyses and warble fly counts. Faecal larval morphological assessment based upon tail morphology and larval size confirmed that *E. rangiferi* larvae dominated the samples (65.8% [56.7–73.7 95% CI], 79/120). *Dictyocaulus* sp. larvae were detected in five individuals (4.2% [1.8–9.4 95% CI]).

*E. rangiferi* was diagnosed as the cause of significant disease in three animals based on clinical signs and histological detection of nematode larva in caudal parts of spinal cord. Histopathological evaluation was performed on most organ samples and severe, chronic granulomatous, verminous pneumonia was found in lungs sampled from two slaughtered animals, likely caused by *E. rangiferi*. Mild interstitial verminous pneumonia was also diagnosed as an additional finding in 17 carcasses. Verminous hepatitis was diagnosed based on histopathological findings in a calf found dead in a slaughterhouse coral. This case of severe granulomatous hepatitis presented with presence of multiple nematodes of unknown identity in liver tissue. The remaining parasitic infections were considered sub-clinical infections. Granulomas in myocardium, diaphragm and liver were found in organ samples from a slaughtered calf. Based on histopathology this was evaluated likely as *Cysticercus tarandi*, the cystic stage of *Taenia krabbei*.

Bone marrow fat index was correlated with some of the identified parasites, with a significant negative correlation for bone marrow fat index with the burden of infection with *H. tarandi* abundance (p < 0.01) and approached significance for the abundance of some gastrointestinal nematodes (*Trichostrongylidae* eggs; p = 0.07 and *Nematodirus* spp.; 0.13).

### Trauma

Trauma was considered as the cause of death in 9% [3.8–13.8] of the carcasses (11/125). Seven animals were hit by car, one was hit by a snowmobile, and three others had fatal traumas with unknown aetiology (diaphragmatic hernia, thoracic stab wound and bone fracture). Trauma was diagnosed in eight adults (3 males and 5 females), two yearlings and one calf (all females) (Fig. 2). The animals were evenly divided between Troms and Finnmark and were in good body condition.

### Diseases related to feeding

Diseases related to feeding were considered as the cause of death in five animals: three animals with ruminal acidosis and two with enteritis due to feeding. One adult was fed with grains from brewery production and developed a catarrhal/haemorrhagic enteritis. Another adult had acute catarrhal enteritis. Bacterial culture showed an overgrowth of *Clostridium perfringens* in the jejunum of both adults, however, *C. perfringens* enterotoxins were not detected.

### Neoplasia and congenital anomaly in organ samples

Neoplasia was found in organ samples from three slaughtered adult females and one calf: T-cell lymphoma in a kidney, pulmonary and hepatic haemangiosarcoma, pulmonary adenocarcinoma and in the calf, multiple bile duct cysts, likely a congenital anomaly in the liver.

### Other

A calf, which died from emaciation in January, had mandibular hypoplasia. An adult female had haemopericardium from an apparent rupture in the left atrium, but of unknown cause. The carcass was in good body condition and there were no signs of trauma. Four dead animals were found on a grass field at the beginning of December. One carcass was submitted for necropsy and histopathology showed an interstitial nephritis with infiltrates of plasma cells and lymphocytes as well as multifocal tubular necrosis indicating some kind of toxic nephropathy. Interstitial nephritis with lymphocyte and plasma cells, was also found in an adult female that had been fed for some time with silage and pellets. Chronic, necrotising hepatitis, with diffuse fibrosis and multifocal large necrotic areas with neutrophils and cell debris was found in a liver sample from a slaughtered adult female. Focal splenic fibrosis was found in a slaughtered calf.

### Unknown

The cause of death was not established in 17 carcasses.

## Discussion

Our study found a wide range of causes of death in reindeer on winter pasture and in calves during their first months of life. The main findings of our study include comparison of disease and predation related deaths to body condition metrics using both the bone marrow fat index [44] and visual fat score. Co-morbidities were seen in a number of diagnoses like bacterial sepsis subsequent to non-fatal predator wounds and poorer body condition with infections.

The comparison of visual fat score and bone marrow fat index correlated well in animals emaciated or in poor condition (index 0–1) as well as in good condition (index 4–5). However, the group of carcasses considered as fair (index 2–3), showed more variation and thus bone marrow fat index alone seems less suitable in this group. The bone marrow method does not distinguish between degrees of good condition (good – very good, levels 4 and 5) and between emaciated to very poor condition levels (levels 0 and 1) since the fat content in bone marrow will be the same [44]. Thus, three categories of fat index, based on bone marrow fat index, seems more appropriate than the five used in the visual fat score. Ideally, the bone marrow fat index should be used in combination with visual fat assessment of the carcass. This has also been the conclusion in other studies that evaluated measures of body condition in reindeer [44, 48].

Emaciation is an important cause of death in ungulates during winter, even if the actual number of deaths are rarely documented. In winters with difficult pasture conditions, the reindeer population can decrease drastically, and emaciation is considered the most likely cause [49, 50]. Winter crises also influence the reproduction in the spring and thus have a negative impact on the number of calves available in the autumn. Calves born from females in poor condition in spring have a reduced survival rate during spring and summer [51, 52], however, by autumn the calves’ condition seems to no longer be related to the mothers’ weight at birth [52]. No winter crises were registered during this study period. Obviously, winter conditions were variable and had, in all likelihood, an important impact on the animals dying from emaciation. However, documenting snow and weather conditions was outside the scope of this project.

Additional feeding was provided prior to death to most of the reindeer that died from emaciation in our study. Some of them had had access to feed for longer periods whilst for others it was only a few days. Emaciation in spite of feeding is not a new issue [37]. Reindeer herders today are better prepared for winter crises with the increased availability of commercial feedstuffs and greater experience with winter-feeding. These animals are, however, still primarily adapted to grazing on natural pastures, which highlights the importance of access to suitable grazing, even when feedstuffs are available.

In our study, 10 animals were considered as emaciated secondary to infectious disease. This highlights that although emaciation can be a result of hunger due to poor pastures or unfavourable snow conditions, other causes such as diseases, high parasite load, injuries as well as stress from predators contribute to nutritional imbalance [53, 54].

Building energy reserves during summer is the key for winter survival in the Arctic and fat metabolism will naturally increase during winter [55]. In our study, the emaciated animals were seen mainly in late winter/early spring, as expected. Young animals are always the most vulnerable having the highest nutritional needs [56], however we did not see any significant age differences between the emaciated animals in our study. This is likely due to bias in the sampling as the motivation for delivering carcasses for inspection in many cases was to confirm suspected predation. In addition, adults are prioritized when searching for lost animals [7] and calves are harder to find since they are quickly eaten by scavengers.

Traditionally, herds had more males and castrates who are able to dig through deep snow and ice, which the females then follow to take advantage of [57]. Females keep their antlers during winter, thus have a higher rank than the bulls, and can chase them away from their grazing holes. One can question whether the herd structure incentivised by public administration policy, which has led to a population predominantly of females and calves, has led to a less robust populations during hard winters [58].

Eagles were the most important predator and responsible for 50% of the total predator killed reindeer. We do not claim that this percentage is representative for Troms and Finnmark, but it is close to the official statistics. However, calves were the main prey for eagles whilst wolverine prayed on both adults and calves is in our study, which is in accordance with public statistics [10].

Reports have also concluded that eagles kill calves predominately during the first months of life [15, 16, 59] and newborn calves with lower body weight seem to be particularly vulnerable to predation [15, 16]. Nieminen et al. [60] also found that calves born from young females, late in the spring, were lighter and had a higher risk of predation. Open landscape seems to be a risk factor for predation of young calves [15, 16]. Eagles are also capable of killing adult reindeer, predominantly by puncturing the chest/thorax with their talons [36, 60]. One adult female was confirmed as killed by eagle in October in our study. Relatively few such cases are reported in the official statistics [10].

Our findings not only confirm that the eagles kill newborn calves but that they also continue to kill calves during the whole first year of the calves life. This is in accordance with official statistics, showing that eagles were responsible for 49% of documented predator killed carcasses in West-Finnmark in 2016–19, with calves being the dominant prey, while lynx and wolverine amounted for 24% and 25% [10]. In our study, the eagle-killed calves were found mainly in the period January-March. This may be a bias of the study rather than a true reflection of the level of eagle predation. These months have light and snow conditions suitable for searching for carcasses.

Our study showed that calves, mostly from the winter pasture in West-Finnmark, were in both poor and good condition when killed by eagles. Josefsen et al. [35] also found that predator killed reindeer had normal body condition. An important aspect is also that poorer body condition is a normal finding in late winter. This might suggest that predator losses in reindeer in Norway are a combination of both compensatory and additive, and not just compensatory losses as suggested by others [17, 39]. An important aspect is also that long-term stress can have negative effects on an individual’s growth, health, and reproduction and this stress should therefore be considered as a negative effect from predators in addition to the fatalities [53, 54]. Assessing the cost of predation and evaluating mitigation measures needs to go beyond the current focus of body condition as a main risk factor and would benefit from a more nuanced approach.

There were few only five killings by lynx in our material. Lynx can kill both adults and calves, but seem to prefer calves [61].

Official statistics from West-Finnmark show documented wolverine kills in 34% of the adults during the herding years 2017/18 and 2018/19 [10]. We found that wolverine killed mainly adults in good body condition (10/14). Some of these adults (n = 3) had non-deadly wounds, but were either euthanized or died subsequently from bacterial infection in association with these non-fatal wounds. It is interesting though that wolverine, despite being a rather small animal, attacks adult males and females in good body conditions weighing 60–80 kg. Killing of adults in a prey population may have a larger impact on the population than predators selecting mainly juveniles [20, 61]. Traditional Sámi knowledge tells about the impact of snow condition on wolverine predation strategies. It describes how wolverine take advantage of deep snow and attack adult reindeer, which find it difficult to escape, as well as sneaking up on reindeer digging grazing pits. Herders have also experienced that reindeer with non-fatal wounds from wolverine would likely die [62]. Bacterial infections in wounds from attacks as seen in a few individuals in our study are likely underreported.

Changing herding practice with supplementary feeding and combination with corralling in particular, as a response to predation or poor grazing availability, can lead to increased risk of infectious diseases [30]. Outbreaks of diseases seems to be an increasing problem in Finland and in Sweden [29, 30], but to a much lesser extent in Norway with only a few published reports [31, 32]. In this project, we did not discover any outbreaks of infectious disease in combination with feeding or corralling.

Infections were, however, still the third most common diagnostic category and were found as the direct, or indirect, cause of death in 14% (20/138) of the individuals in our study. Emaciation was also seen in combination with infections and bone marrow fat index was significantly lower in animals dying of infections than of trauma, or of other causes. Infections can lead to general weight loss [63] and reindeer, being in a negative energy balance during winter [64], will be particularly vulnerable to emaciation if exposed to disease. On the other hand, animals in poor condition also have increased susceptibility to infections [65].

Infection with *P. multocida* was the dominant infectious disease in the project. Pasteurellosis is a known disease in semi-domesticated reindeer with a potential for large outbreaks [66] but probably underreported, since single cases and smaller outbreaks seldomly are diagnosed. Acute infections can affect animals in good condition, often in combination with stress, as seen in our data when calves in good condition died of acute pasteurellosis. Septic pasteurellosis was diagnosed in four calves; three were found dead after transport at the same slaughterhouse coral, whilst one died during transport. Bronchopneumonia due to *P. multocida* was found in a calf with rich fat reserves that died after showing clinical signs of respiratory disease. The bacteria were also found in individuals with septicaemia subsequent to a predator attack. In two of the cases, adults were found dead with non-fatal bite wounds caused by a wolverine. In another case, an adult reindeer was euthanized after being found alive with bite wounds from a wolverine. The fourth case was in a young calf diagnosed as killed by lynx. Bacterial septicaemia in combination with the predator attack could have been due to activation of the animal’s own mucosal bacteria because of severe stress from being chased and attacked. The other explanation is the transfer of the bacteria from the predator’s oral cavity with a bite wound infection. Bite wounds from dogs and cats are the most common cause of infections with *P. multocida* in humans [67].

Other bacterial infections included three carcasses from which *T. pyogenes* was isolated. One calf had embolic pneumonia with multifocal inflammation dominated by neutrophils and macrophages and generalised bacteraemia, whilst an adult had purulent peritonitis caused by a perforating wound into the dorsal part of the abdominal cavity. Such puncture wounds could have been caused by an eagle attack, but this was not possible to document. The third case was a yearling with purulent arthritis. *Streptococcus* sp. was isolated from another calf with osteomyelitis, arthritis, and periarthritis, and *Streptococcus suis* was isolated from a liver abscess in an adult. *Moraxella* sp. was isolated from the eyes of two carcasses with mild purulent conjunctivitis and from a live animal with keratoconjunctivitis. The latter case was part of a small outbreak of eye infection in a herd during transport to winter pasture. Other bacterial diagnoses include beta-toxin producing *Staphylococcus* sp. isolated from skin abscesses in the axillae of an adult and *E. coli* from a two-week-old calf found dead with acute, catarrhal enteritis.

Although elaphostrongylosis was diagnosed in only three calves from Trøndelag, these three cases were part of a large outbreak which included the loss of approximately 150 animals over a period of two years after an unusually warm summer and autumn in 2018 [68]. Single cases of elaphostrongylosis are regarded as common and seldom reported, but larger outbreaks have been documented. Outbreaks generally occurred in late autumn/early winter after unusually warm summers and the impact of temperature is well documented [33]. Elaphostrongylosis can also have milder, unspecific symptoms such as confusion, unusual tameness, lethargy and weight loss [33].

We also found mild meningitis in a yearling that died from ruminal acidosis. This yearling had withdrawn from the herd and stayed close to a house, which could be symptoms consistent with elaphostrongylosis.

Handeland et al. [48] found that high number/load of the three parasites *C. trompe,* *H. tarandi* and* E. rangiferi* in wild reindeer influenced the body condition in calves and increased the risk of emaciation [48]. However, the mean load/range of these parasites were much higher than those seen in our results. Despite this, we also saw, that animals with higher *H. tarandi* burdens had significantly lower bone marrow fat index than those with lower burdens or no warbles. Infections with endoparasites were common in our study but mainly without pathologic changes or reduced body weight.

We identified a calf with mandibular hypoplasia. This type of malformation is known to occur sporadically in reindeer [36]. One would expect such calves to have trouble with suckling as newborn and that they would die soon after birth [35]. However, this calf had managed to feed for 8–9 months before it perished.

The results from this study are not representative for specific regions or districts. Most of the material came from the winter pastures of inner Finnmark. The carcasses and bone samples were mostly from material delivered to SNO for examination for predator damage/wounds. The organ samples were from animals that had either died before slaughter or were random findings during meat inspection at slaughterhouses. In Troms, the carcasses were mostly delivered when predator kills seemed unlikely. The month of submission varied with most carcasses being submitted during winter and early spring (December-May) except for a few newborn calves, which were submitted in spring/early summer (May–July). The period during which the vast majority of carcasses were submitted (January-April) is the part of the winter with the best light and weather conditions for locating carcasses, and the cold weather also helps to preserve the carcasses. The few carcasses from Trøndelag were animals delivered due to clinical symptoms of brainworm infection. This makes a comparison of geographic and temporal trends challenging and was therefore not appropriate for this dataset.

## Conclusions

This study is descriptive and shows current causes of death and disease and the context of body condition at the time of death. We found that predators killed animals in both good and poor condition during winter and emaciation was sometimes associated with infections. Even if loss of animals can be related to assumptions such as climatic conditions, quality of the pastures and the presence of predators, losses are also a result of unforeseen events. Causes of loss are often complex, and the study shows the importance of examining the animals that die.

## Data Availability

The dataset used in the current study is available from the corresponding author on reasonable request.
